# Study on Instant Delivery Service Riders' Safety and Health by the Effects of Labour Intensity in China: A Mediation Analysis

**DOI:** 10.3389/fpubh.2022.907474

**Published:** 2022-06-23

**Authors:** Tianxue Chen, Dazhou Tian, Peihua Deng, E. Zhou, Jinjin Huang

**Affiliations:** ^1^School of Public Administration, Zhongnan University of Economics and Law, Wuhan, China; ^2^Chinese Academy of Labour and Social Security, Peking, China

**Keywords:** IDS riders, labour intensity, safety and health, instrumental variables, mediating effect, moderating effect

## Abstract

The Instant Delivery Service (IDS) riders' overwork by “self-pressurisation” will not only reduce the level of their physical and mental health but also lose their lives in safety accidents caused by their fatigue riding. The purpose of this article is to examine whether there is overwork among IDS riders in big and medium cities in China? What's going on with them? Based on the Cobb-Douglas production function in the input-output theory, this study characterised the factors on IDS riders' safety and health associated with labour intensity. A mediating model with moderating effect was adopted to describe the mediation path for the 2,742 IDS riders who were surveyed. The results of moderating regression demonstrated that (1) 0.4655 is the total effect of labour intensity on the safety and health of IDS riders. (2) 0.3124 is the moderating effect that working hours make a greater impact on labour intensity. (3) The mediating effect of work pressure is the principal means of mediation both upstream and downstream.

## Introduction

The Instant Delivery Service (IDS) riders have been linked to the city's capillaries. The outbreak of COVID-19 prompted the number of IDS riders to increase in early 2020. The IDS riders delivered food, vegetables, and medicines to the inhabitants ignoring the dangers to themselves both day and night during the epidemic period. 20.8% of IDS riders are serviced by riding more than 50 kilometres per day (1). The number of workers has increased significantly in new employment forms such as online appointment distributors, online appointment drivers, and Taxi drivers employed on the Internet platform. IDS workers have bounded to the “human component” (2) of IDS which set up the game mechanism depending on the AI system, especially the IDS riders.

Most of the work of couriers takes place outdoors, where they are exposed to various environmental conditions such as weather, pollution, and the risk of accidents (3). The demand for fast deliveries and payment per delivery in some modes of employment put extra stress on couriers that increase the risk of unsafe behaviours and involvement in accidents (4–6). Statistics for Great Britain show that motorcyclists are more at risk of being killed or injured in a road traffic crash than any other type of vehicle user (7).

There was a definite relation between hours of work, fatigue, and involvement in a road accident (8). A number of studies have demonstrated that the risk of a motorcyclist having a crash increases with exposure and falls with age and riding experience [e.g., (7, 9–11)], increase crash risk include riding too fast e.g. (12–14).

Gig's work led some couriers to experience impairment caused by fatigue and pressure to violate speed limits and to use their phones whilst driving (15). Descriptive analysis for the assessed mobile phone use while driving (MPUWD) behaviours showed that 96.3% (*N* = 315) of food deliverymen undertook the MPUWD behaviours, food deliverymen, interact with mobile phones while on work-related travels mostly for business rather than entertainment (16). One notable exception was intersections, where the risk of being involved in a conflict was twice as high for e-bikes as for conventional bicycles. The speed immediately preceding a conflict was higher for riders of e-bikes compared to conventional bicycles, a pattern that was also found for mean speed (17). Motorists tend to accept smaller gaps in crossing situations in front of an oncoming e-bike compared to a bicycle approaching at the same speed (17). This effect was hypothesised to be the result of an apparent mismatch between the cyclist's actual speed and the speed perceived by the motorist. Given that the motorised component eases acceleration for the e-bike rider, it could be expected that mis-judgements of e-bike speed are especially prevalent at intersections, resulting in an increased number of conflicts (17). Safety attitudes had a significant negative effect on aberrant riding behaviours. E-bike riders reporting more errors and aggressive behaviours were more prone to at-fault accidents involving. E-bike riders who had stronger positive attitudes towards safety and showed more worry and concern about their traffic risks tended to be less likely to engage in aberrant riding behaviours (18). The emergence of the gig driver could give rise to a perfect storm of risk factors affecting the health and safety not just of the people who work in the economy but of other road users (15). Higher traffic violations of laws, more and more frequent traffic accidents and casualties, it cannot be underestimated for IDS riders' safety.

Today, anyone who can prove their right to work in the UK can put themselves to work via on-demand platforms such as Deliveroo (19), which has successfully drawn in full-time and part-time riders, including students, migrant workers, and those looking to supplement their incomes with gig work (20). Were any of these individuals to suffer a crash, however, they would not qualify for employee compensation. Deliver riders are classified as “self-employed contractors rather than as employees of the company” (21).

IDS companies control the employees by carrying out “over the horizon management” and flat management by Internet technology (22). It is naturally and constantly weakened for the formal employment contract of IDS workers. IDS workers with informal employment relations cannot be protected by China's industrial injury insurance. The amount of compensation is very limited even if parts of workers insure themselves commercially.

The properties of the hegemonic factory regime make the employees working on the IDS platform be no sense of commitment and identity to their profession (23). IDS riders' work is instrumental and transitional. “No life, but work,” (24, 25) The poor living conditions are only for the reproduction of physical strength and recovery of labour tools. IDS rider's concept of labour time and space runs counter to the presupposition of labour law, and their injury to occupational health and physical and mental health deviates from the labour legal standard.

Reportedly, delivery riders are temporarily employed, poorly paid, and often paid “by the job,” e.g., paid by the hour or the number of delivery goods (26). This tends to induce an intense work pace for long hours, without breaks but also higher work stress, work fatigue, and unsafe driving behaviours, e.g., running a red light or a stop sign (27, 28). Since the motorcyclists could be forced by employers to shorten the delivery time (29), they could be forced to commit traffic violations inevitably (27). Many restaurants in Korea maintain quick-delivery service programs to satisfy customers. This service allows delivery workers limited time to deliver, which frequently puts them in danger (30). Stress and work overload were associated with reduced safety behaviour and increased risk of involvement in accidents (31).

The workers' fatigue savings formed by their high intensity of labour and pressure of life seriously affect their safety and health. It is seen often enough that overwork and sudden death of IDS riders.

Papakostopoulos and Nathanael (32) reported that the delivery industry lacks a safety culture, thus making risk-taking acceptable for a delivery rider in Greece. At least in this particular socio-cultural context, the estimated compliance of food delivery companies to safety and health rules suggesting to do so. This is supported by the vast majority of respondents (83%) reporting an intensive work pace (more than 3.5 deliveries per hour) suggesting that employers vastly promote fast delivery over self-protection (32). The fatigue savings of IDS riders mainly arise from the work game design of the platform and the cognitive psychology at work (33–35). To study the association factors and mechanism why IDS riders will be overworked, we should consider the factor of labour intensity. This paper analyzes the mediation path of the impact of labour intensity on safety and health in physical and mental, based on the hypothesis that labour intensity has a direct impact on IDS riders' safety and health.

It is the scholars abroad who first began their research of IDS riders. However, most of the research results focused on the safety guarantee of riding. With the development of China's platform economy, domestic scholars began to study IDS riders. However, most of the research results are related to their life and work on rights (36). In terms of research methods, qualitative research is generally carried out from the perspective of law and management. In these qualitative studies, there are few results on IDS rider health issues. From the literature search, there are few empirical studies on IDS riders by Chinese scholars (37, 38). Of course, little empirical research is on the health issues of IDS riders.

This research mainly makes breakthroughs from two aspects: (1) In discussing the economic problem of IDS riders' income, their safety and health are also considered, and high-quality employment is promoted from the employment problems. (2) An empirical method is used to describe the intermediary path of IDS riders' safety and health.

## Methods

### Participants and Procedure

From June 2020 to June 2021, A total of 3,000 questionnaires were distributed in 16 districts and 2 counties in Beijing and 13 administrative regions in Wuhan (no survey was conducted in 6 functional areas).

The participants of IDS riders are from 12 IDS companies including Meituan, ELM, SF Express, EMS, JD, YTO, STO, ZTO, Rhyme Express, TTK Express, Best Express, and Homestead Express. These 12 companies have the largest scale in China's express industry and absorb a large number of IDS riders. They are also the most standardised enterprises which carry out the labour law standards. For IDS riders, these companies with formal management should be able to better protect their health and avoid overworking. If the employees of these companies are in a state of overwork, it shows that the sample is more persuasive.

According to the “Code of Occupational Classification of the People's Republic of China” (2015 Edition), the “National Occupational Standard for Express Operators” (Draught), IDS riders were identified as our research object. They mainly focus on food and beverage distributors and express delivery. The research in the express industry is usually conducted when they pick up and pick up goods from 9 to 11 a.m. Because their personnel is relatively concentrated at this time. IDS riders in food and beverage distributors usually concentrate from 8:30 to 10:30 am. Before and after the morning meeting of the company, they can communicate freely or inquire about orders online for a while. Because there are few orders during this period, it is more convenient to investigate IDS riders in food and beverage distributors during this period.

In this study, if IDS riders are directly probability sampled, they would refuse to investigate because of their busy work. The sample selection process is judgmental sampling. We get the cooperation of enterprise management through the intervention of the administrative organisation. Judgment sampling, a non-probability sampling procedure, has low operation cost and is also suitable for the objective situation of tight funds in this study. Although there are disadvantages of tendentious influence, IDS riders have a high coincidence of working properties, so the non-probability sampling results can be inferred as a whole.

Among the questionnaires, 2,000 were distributed in Beijing, 1,000 were distributed in Wuhan, 2,848 were recovered, 107 invalids were excluded, and the rate of recovery was 94.9%, and the rate of effectiveness was 91.4%. Of the 106 invalid questionnaires, 95 were incomplete (the IDS riders did not complete the questionnaire). Because the answer results should be analysed together with the working conditions of the respondents, it is necessary to avoid mutual consultation when filling in together in the workplace. Six questionnaires cannot be counted due to the lack of data or inconsistencies between filling in and out. Five questionnaires were filled in by others instead, so we eliminate them. Therefore, we get 2,742 observations.

We take Beijing and Wuhan as the research areas, mainly because Beijing is a megacity and Wuhan is a megacity^1^.

These two cities are very developed and representative cities in express delivery. One is the capital, whose industrial development plays the role of a wind vane. Another reason is that Beijing has a simple terrain of the traffic route map, which can be used as a concise sample representative of urban planning. Wuhan is located in the central region, and its development level is equivalent to the national average standard. Wuhan has complex terrain, intricate, and intersecting rivers, lakes, and a vast area, which can be used as a sample representative of complex urban planning.

### Data Analyses

The questions about safety and health in Part 1 of the questionnaire are mainly based on the Cumulative Fatigue Symptoms Index (CFSI) (39, 40), Fatigue Scale-14 (FS-14) developed by Japan (41), and Fatigue Assessment Instrument (FAI) (42).

Japan's Fatigue Assessment Instrument has many design items, including not only physical but also spiritual. In addition to working conditions, it also analyzes the main causes of damage to health in working life from the aspects of living time and living conditions (43).

The results of factor analysis are classified and a new item classification is obtained^2^. CFSI includes five major causes, including sleepiness (Group V), restlessness (Group V), unhappiness (Group III), fatigue (Group V), and vertigo (Group V). It is composed of 25 subjective fatigue descriptions.

Fatigue Scale-14 was jointly compiled by Trudie Chalder (41) of King's College Hospital's psychological medical research laboratory and G. Berelowitz of Queen Mary's University Hospital in 1992. The scale consists of 14 items, including two dimensions: first, physical fatigue, which mainly evaluates physical strength, muscle strength, and rest, with a total of 8 items; The second is mental fatigue, which mainly evaluates memory, attention, and quick thinking, with a total of 6 items. FS-14 requires respondents to answer “yes” or “no” according to their actual situation, in which “yes” is 1 and “no” is 0. The higher the score, the more serious the fatigue is.

Fatigue Assessment Instrument was formulated by Joseph E, Schwartz of the American Psychiatric and behavioural sciences research laboratory, and Lina Jandorf of the neurology research laboratory in 1993 (44). Workers can make self-assessments based on this scale, which includes four dimensions: first, the severity of fatigue, which has 11 items; Second, the environmental specificity of fatigue, including 6 items; The third is the result of fatigue, including three items; fourth, the response of fatigue to rest and sleep, including 2 items. Each item in FAI shall be graded from 1 to 7.

The scale consists of 14 items, including two dimensions (45): first, physical fatigue, which mainly evaluates physical strength, muscle strength, and rest, with a total of 8 items; The second is mental fatigue, which mainly evaluates memory, attention, and quick thinking, with a total of 6 items. Fs-14 requires subjects to answer “yes” or “no” according to their actual situation, in which “yes” is 1 and “no” is 0. The higher the score, the more serious the fatigue is.

In the questionnaire of IDS riders, we designed options such as irritability, unable to control emotions, sleepy at work, lack of motivation, feeling weak limbs, and so on. According to the data in the survey, 50.4% of IDS riders sometimes felt impatient, irritable, and unable to control their emotions by themselves. They sometimes want to drop express items on the ground. 20.2% of them even often did that too. In the options about health, 27.2% of them have frequent physical issues (headache/dizziness/heart discomfort/tinnitus/dizziness, etc.). Comparing their stress of mental with physical, 56.3% chose “mental stress is greater than physical stress.” Regarding work status, 30.6% were in the range of 9–20 (II). 42.5% were in the range of 21~27(III). More than 20.4% were above 28 (IV) in the statistic. Making statistics on work Burden Indices, we found that 26% were in the early warning zone, 35.1% were in the danger zone, 23.3% were in the danger zone, and 6.2% were in the high-risk zone.

From the analysis above, according to the statistical data, we would conclude that most IDS riders were in an overworked state.

### Variables

#### Dependent Variable

The dependent variable is the Work Burden Index reflects the degree of overwork in the zone of health risk, which is to measure the status of IDS riders' safety and health. The variables are in order. A higher value indicates lower health. The Work Burden Indexes representing safety and health are classified by I, II, III, and IV levels based on the 2 scales of self-conscious symptoms and working conditions.

The 7 points in a matrix^3^. form different zones of safety and health. The safety zone, early warning zone, dangerous zone, and high-risk zone can be seen in Table 1.

**Table 1:** Score of IDS riders' overwork (Work Burden Index).

**Self-conscious symptoms**	**Working status**			
	**A**	**B**	**C**	**D**
I	0 (safety zone)	0 (safety zone)	2 (early warning zone)	4 (dangerous zone)
II	0 (safety zone)	1 (safety zone)	3 (early warning zone)	5 (dangerous zone)
III	0 (safety zone)	2 (early warning zone)	4 (danger zone)	6 (high-risk zone)
IV	1 (safety zone)	3 (early warning zone)	5 (danger zone)	7 (high-risk zone)

*Source: Based on the comprehensive judgment of “CFSI, FS-14, FAI.”*

In the zone of 0–1 point, IDS riders' job is easy and the hours are good; In the range of 2–3 points, IDS riders have a work burden, and their safety and health in the early warning zone; In the range of 4–5 points, IDS riders have a higher work burden and be in the danger zone; In the range of 6–7 points, IDS riders are in the high—risk zone.

According to the Work Burden Index matrix, safety and health are mainly completed in three steps.

Step 1: Divide the fatigue symptoms into health grades according to the Self-Diagnosis Scale of Fatigue Accumulation of Workers issued by the Ministry of Health, Labour, and Welfare in Japan, Safe Production Law of the People's Republic of China, and Guiding Opinions on Safeguarding Workers' Rights and Interests of Labour Security in New Forms of Employment issued by eight departments in China^4^. Construct an evaluation system to measure IDS riders' work burden. This system combined with the Fatigue Assessment Instrument, FS-14, Fatigue Scale-14, and the ten early warning signals of “overwork death” issued by the Japan Overwork Death Prevention Association. Then a scale is established to measure IDS riders' subjective feelings of fatigue, including 14 items: such as “impatient, irritable, unable to control their emotions, sometimes want to drop the delivery items on the ground,” “frequent physical issues (headache/dizziness/heart discomfort/tinnitus/dizziness, etc.).” The scores and grades are as follows: 0–8 is for I, 9–20 is for II, 21–27 is for III, and 28 or more is for IV.

Step 2: Classify the grade of work level. Eight items are in the work condition evaluation form, such as “sudden increase in the number of receiving and sending, the need to work overtime” and “mental pressure caused by work.” The standard of score and level are: 0 ~ 8 points are Grade A, 9–15 points are Grade B, 16–23 points are grade C, and more than 24 points are grade D.

“Five score rating scale” was drawn according to IDS riders' work. They are “never so (0), rarely so (1), sometimes so (2), often so (3), and always so (4).”

Step 3: Build a conscious symptom and work condition matrix to determine the safety and health zone of IDS riders.

#### Independent Variables

According to National Standard GB3869-83, physical labour intensity was divided into four levels: I (light labour), II (medium labour), III (heavy labour), and IV (extremely heavy labour). The measurement indicators are average energy consumption and net labour time^5^. However, it is difficult to measure the energy consumption in the survey. IDS riders' work is mainly determined by working hours and riding distance. (In the structural survey, we found that many riders are not only interested in the number of orders, but also concerned about their daily odometer). In this paper, the “average daily distance” is instead of the “energy metabolism rate” in National Standard GB3869-83. We keep the calculation formula and calculation coefficient unchanged. A calculated six-level labour intensity index is used as the independent variable.

#### Instrumental Variable

As the needs of the instrumental variable are correlated with the independent variables, the frequency of traffic offences and orders are tailored to the instrumental variables. The correlation is that IDS riders are easily distracted while driving in the mood of high work intensity and pressure. It is easy for IDS riders to cause traffic violations facing plenty of orders. Labour intensity increases with the increase in the number of orders. There is a correlation between the number of orders and labour intensity.

The instrumental variable also needs to meet the condition that it is not correlated to the disturbance item. We find that the number of orders is qualified as an instrumental variable that has no correlation to the random disturbance term. However, being the new economic form, IDS riders obtain tasks randomly on the platform through “online order-grabbing.” Random orders are knocked out without limited time and placed by different millions of customers on the platform. Since the random orders do not correlate with the error term, the instrumental variable is credible.

Another instrumental variable of traffic violation frequency has random too. Complex and changeable traffic full of the constant flow of vehicles and people randomly happens on traffic violations or accidents. Similarly, traffic violation frequency meets the requirements of an instrumental variable.

### Theory Model

An increasing number of employees of IDS workers sign up for the IDS platform. It is a rational choice for flexible employees under the development situation of the new economics. U_*ij*_ is for the utility of the IDS riders in the state of reasonable working hours and pleasant mood, and *U*_*ik*_ is for the utility while they are in physical and mental fatigue state. WhenU_*ij*_≥*U*_*ik*_, IDS riders have higher physical health. IDS riders' utility is determined by career and restricted by the status of their family economy and the way to get income. As it is known to all, those characteristics of IDS riders have a low employment threshold, which makes them limited space for career to transform and less income to improve. An Intertemporal Utility Model for IDS riders can be constructed using the C-D Function:


$$\begin{array}{l} U={\text{L}}_{\text{1}}^{\theta }{\text{L}}_{2}^{1\text{-}\theta } \end{array}$$

Where U is for the utility of IDS riders, L1 and L2 are for IDS occupation and leisure consumption while quitting IDS, θ∈[0, 1].

Suppose that labour intensity is α∈[0, 6]^6^. In this paper, the average daily distance is used to replace the energy metabolic rate in the National Standard GB3-83. We keep the calculation formula and the coefficient of the labour intensity index. The “Daily Distance” is divided into 6 levels as the labour intensity index.

A rise α indicates an increase in labour intensity. The budget constraints for the two periods are


$$\begin{array}{l} {\text{L}}_{1}+\text{C}+\text{S}+{\text{B}}_{1}\alpha \leq \omega (\alpha )t(\alpha )+\text{E}\\ \;{\text{L}}_{2}+{\text{B}}_{2}\alpha =\text{Y}+\lambda \text{S-}\ln f \end{array}$$

Among them, L_1_is the labour supply during the working period, and C is the total consumption expenditure for IDS riders' daily living; S is the savings during the working period, is the health cost during the working period, is the salary, *t*(α) is the working hours, and E is the non-labour income. The labour intensity α will affect by health costs, wages and working hours, etc. L_2_ is the consumption of physical or leisure while quitting IDS, refers to the health cost after quitting IDS occupation, Y refers to the IDS riders' old-age pension, λS refers to the savings and transferred interest after quitting IDS occupation, and ln *f* is the legacy and death gratuity left to their family. Consumption capacity after they quit IDS depends on their all pension, savings, and the property left to their family.

Construct the Lagrange Function and take a derivative of labour intensity to solve IDS' Intertemporal Utility Function:


$$\begin{array}{l} \frac{\partial \text{U}}{\partial \alpha }={\left[\lambda (1-\theta )\right]}^{1-\theta }\theta^{\theta }[\frac{\partial \omega (\alpha )}{\partial \alpha }t(\alpha )+\frac{\partial t(\alpha )}{\partial \alpha }\omega (\alpha )-\\ \;\left(\frac{\partial B_{1}(\alpha )}{\partial \alpha }+\frac{1}{\lambda }\frac{\partial B_{2}(\alpha )}{\partial \alpha }\right)] \end{array}$$

It can be seen from the above formula that the impact direction of labour intensity on the occupational utility of IDS riders is related not only to wage and working hours *t*(α), but also to the impact of labour intensity on wagesand working hours$\frac{\partial t\left(\alpha \right)}{\partial \alpha }$, and so does the impact on intertemporal health costs and .

### Measurement Model

The average age of IDS, riders is 26.4 years old. The employees are mainly male youth. This paper assumes that IDS riders are homogeneous, [λ(1−θ)]^1−θ^and θ^θ^ are constants^7^ and >0; More labour supply will result in increases in working hours and salary. Both $\frac{\partial \omega \left(\alpha \right)}{\partial \alpha }t\left(\alpha \right)$ and $\frac{\partial t\left(\alpha \right)}{\partial \alpha }\omega \left(\alpha \right)$ will be >0. IDS riders will ignore or overdraft the health cost (current and future) to work which is likely an economic rational choice under the constraint of the platform. and will be also >0. According to these theories, we can deduce Hypothesis 1.

H1: The increase in riders' labour intensity makes the Work Burden Index rise, which eventually leads IDS riders to overwork and is in the zone of health risk.

Due to the order-grabbing system in the IDS industry, the substitution effect of labour supply caused by salary is greater than the income effect. To increase working hours, the result is to improve labour intensity. From this, we can get Hypothesis 2.

H2: Salary and working hours play a moderating role.

The effect of increasing work intensity on health is not direct, where job stress and job satisfaction have a mediating role. Therefore, we can propose Hypothesis 3.

H3: Job stress and job satisfaction play a mediation role between labour intensity and safety and health.

Labour intensity is ordered by multiple categorical variables, and its value has only ordered significance and lacks an interval scale. Therefore, this study selects the Ordered Multinomial Logistic Regression Model:


$$\begin{array}{l} {\text{H}}_{ij}=\alpha +\beta LI_{ij}+\gamma {\text{X}}_{ij}+\varepsilon_{ij} \end{array}$$

Where, H is the health degree of IDS rider *i* in an urban area *j*, and *LI* (Labour Intensity) is the labour intensity of IDS rider *i* in an urban area *j*; X_*ij*_ is the control variable group; α, β and γ are the parameters to be estimated.

However, there are many factors affecting the health level of IDS riders. In addition to the physical health level characteristics of IDS riders, there are also external complex factors such as urban discrimination against migrant workers, riders' living conditions, and living environment. It is difficult to completely control in the model, and other unobservable factors may be omitted, which would result in the endogenous problem. On the other hand, there can be a reverse causality between labour intensity and safety and health. Different knowledge about familiarity with urban rods will make IDS riders safer and healthier. The safety and health of IDS users will also be exacerbated by the degree of congestion in various sections of urban traffic and psychological discouragement after poor evaluation. Due to the endogenous problem, this document selects an identification strategy by the instrumental variable analysis. We adopt IV-Logit Two-stage Estimation Method after adjusting the Logit Model above.

## Results

### Estimated Results

The regression results in Table 2, Model 1 only includes the core independent variable, and the significance of the regression results is not too strong. The age control variable was added to Model 2, and the test results were slightly improved. Three control variables of age, education, and gender were added to Model 3, and the significance was greatly enhanced. Model 4 adopts the IV-Logit Two-stage Method, the regression coefficient is more statistically significant, and the result is more reliable.

**Table 2:** Model estimation results.

	**Model 1**	**Model 2**	**Model 3**	**Model 4(IV)**
LI (labour intensity)	0.0054* (0.0645)	0.0263* (0.0232)	0.3723** (0.0406)	0.4655*** (0.0308)
Age	-	0.5766** (0.1433)	0.4725* (0.4407)	0.6724*** (0.3423)
Academic	-	-	0.2724* (0.1425)	0.2215** (0.3087)
Gender	-	-	-	0.1212 (0.5013)
Instrumental variable	-	-	-	0.3016** (0.4371)
*R* ^2^	0.432	0.397	0.365	0.412
Adjusted R-square	0.7378	0.6162	0.5164	0.7188
_F_	23.56***	25.77***	28.32***	22.66***

***P ≤ 0.001, **P ≤ 0.01, *P ≤ 0.05; Robust standard error in brackets.

*Source: authors' own*.

In model 4 (IV), the regression coefficient is significantly positive and the intensity is high. H1 is verified and supported. The increase in labour intensity will greatly increase the work burden, so it is a negative impact on safety and health.

### Extreme Working Environment Analysis

We did not do a heterogeneity test for IDS riders of the model. The safety issues, more dangerous than health, are caused by the extreme working environment. Structure investigation is adopted to analyse the problem of safety.

Instant Delivery Service riders are exposed to outdoor work most of the time. The external factors harm their safety and health, such as hot sun, high temperature, fierce wind, rain and snow, and daily breathing exposure. Daily breathing exposure is mainly due to the excessive PM2.5 in haze air. The little difference in daily breathing exposure for the whole urban residents, so IDS riders are afraid of the hot sun, high temperature, wind, rain, and snow. Poor working environment, especially the extreme weather has the greatest impact on IDS riders. However, in the survey, it is believed that the impact of wind, rain, snow and high temperature, and hot sun on safety are, respectively, 78, 88, 94, and 23%. According to the structural interview survey, IDS riders generally don't care about the high temperature and hot sun. What is the reason that they take the first three items seriously, but the high temperature? It may be that the high temperature cannot prevent them from delivering. Another reason may be that they know little about the probability of heatstroke which leads to death. They have no medical understanding that high-temperature thermal fatigue may potentially induce safety and health issues, such as heart disease and coronary heart disease.

### Mediating Path Analysis

The transmission path between labour intensity and safety and health is as follows: the stronger become the labour intensity, the greater cost the of safety and health. Labour intensity is not beneficial to physical safety and health.

A high income formed by labour utility may make IDS riders satisfied with their work. High satisfaction work makes workers be in good health, physical and mental. The impact is positive. While the work pressure of long working hours does harm workers physically and mentally. Then, that impact is negative. Therefore, in this study, we take income and working hours as moderating variables.

Salary has a substitution effect and an income effect. The income effect makes fewer working hours, while the substitution effect makes workers work more. The total effect is uncertain. But for IDS riders, being a game of orders-grabbing, the substitution effect of salary is greater than the income effect.

Our study explores the variable's role and the mediation path by applying Muller's Chain Mediating Effect Model with moderating variables.


$$\begin{array}{l} \;{\text{H}}_{ij}=\alpha_{1}+\beta_{1}LI_{ij}+\varphi_{1}G_{ij}+\vartheta_{1}LI_{ij}\text{*}G_{ij}+\gamma_{1}X_{ij}+\varepsilon_{1}\\ \;{\text{M}}_{\text{p}}=\alpha_{2}+\beta_{2}LI_{ij}+\varphi_{2}G_{ij}+\vartheta_{2}LI_{ij}\text{*}G_{ij}+\gamma_{2}{\text{X}}_{ij}+\varepsilon_{2}\\ {\text{H}}_{\text{ij}}=\alpha_{3}+\beta_{3}LI_{ij}+\varphi_{3}G_{ij}+\vartheta_{3}LI_{ij}\text{*}G_{ij}+\phi_{3}M_{p}+\tau_{3}M_{p}\text{*}G_{ij}+\gamma_{3}{\text{X}}_{ij}+\varepsilon_{3} \end{array}$$

H_*ij*_ represents safety and health, and G_*ij*_ represents the moderating variables of salary and working hours^8^ The data on salary comes from the 7-income range of monthly income in the questionnaire, and the data on working hours come from 4-time ranges in the questionnaire.

M_p_(p =1-3)is the mediating variable: work stress M_1_includes 5 dimensions, Calculate the mean value of 5 dimensions as the variable: “24 h rest/month, labour contract, social security, perception of competitive stress, and poor comments,” which is answered in four grades. Job satisfaction M_2_ is investigated from six dimensions. The mean value is calculated in the same way. Six dimensions are “organisation satisfaction, management satisfaction, job reward satisfaction, working atmosphere satisfaction, the job itself satisfaction, and human resource management satisfaction,” which are answered in five levels of satisfaction. The chain mediating variableM_3_ is expressed by “job stress ^*^ job satisfaction.”

### Mediating Effects Test

To analyze the mediating effect neatly, the moderating effect is not considered.

According to the mediating effect results in Table 3, the front-end effect of Road 1 is significant, and the back-end effect of the Road 1 mediation path is also very significant. It is not difficult to find that job pressure is the leading mediating variable leading to safety and health. Hypothesis 3 is verified job pressure has a mediating effect.

**Table 3:** Estimation results of chain mediating effect.

**Mediation path**	**Total effect β_1_**	**β_2_**	**ϕ_3_**	**Direct effect β_3_**	**Mediating effects β_1_-β_3_**
Road1	0.4655*** (0.0308)	0.0076*** (01352)	0.0063** (0.5643)	0.3093** (0.1338)	0.1562*** (0.5437)
Road2	0.4655*** (0.0308)	−0.0021 (0.2322)	−0.034*** (0.9321)	0.4668 (0.7985)	−0.0013* (0.3428)
Road3	0.4655*** (0.0308)	0.0016 (0.5323)	−0.027 (0.1421)	0.4913* (0.5363)	−0.0258** (0.5712)
LLCI	0.2763	0.1242	0.0322	−0.1426	0.2152
ULCI	0.5734	0.3216	0.0453	−0.1043	0.4026
*R* ^2^	0.348	0.235	0.350	0.359	0.294
Adjusted R-square	0.4823	0.5326	0.5035	0.6011	0.6202

***P ≤ 0.001, **P ≤ 0.01, *P ≤ 0.05; Robust standard error in brackets.

*CI, confidence interval. Source: authors' own*.

### Moderating Effect Test

Data is centralised before the two variables G_*ij*_are used in moderation. Hierarchical Regression is carried out for the two generated interactive variables. The linear regression estimation results of the model with moderating variables are shown in Table 4.

**Table 4:** Estimation results of moderating effect.

**Variables**	**Model 1**	**Model 2**	**Model 3**
Salary level	0.0246 (0.6456)	0.3524 (0.1432)	0.2636* (0.4365)
Labour intensity * salary level	0.0124 (0.4328)	0.0324** (0.6834)	0.1537* (0.7156)
Working hours	0.5265 (0.9342)	0.4278 (0.8326)	0.5023*** (0.2475)
Labour intensity * working hours	0.2146** (0.3215)	0.3547*** (0.5452)	0.3124*** (0.3486)
Age	-	0.5375 (0.7246)	0.4328*** (0.6143)
Academic	-	-	0.2321 (0.4122)
Gender	-	-	0.1326* (0.5129)
*R* ^2^	0.4023	0.2011	0.3212
Adjusted R-square	0.6782	0.5163	0.6166
VIF	3.135	2.242	3.026

***P ≤ 0.001, **P ≤ 0.01, *P ≤ 0.05; Robust standard error in brackets.

*VIF, variance inflation factor.*

*Source: authors' own*.

According to the estimation results of the moderating effect in Table 4, the impact of salary on labour intensity is not significant, and the extension of working hours has a positive effect on labour intensity. The significance test of multiplication of salary and labour intensity is passed at 10%. Though the moderating effect of salary on labour intensity is determined by the income effect and the substitution effect, the total effect is positive. Facing the temptation of salary, IDS riders will take the initiative to grab more orders to earn more money. In reality, platform work has only changed mechanisms through which companies can exercise control over labour and evade their employer obligations. The freedom of food delivery platform workers is essentially an “illusory freedom” (49).

As a result of an increase in labour intensity, they will be overworked, which is no benefit to their health. The multiplication of working hours and the labour intensity is tested at the significance of 1%. It shows that the IDS riders' labour intensity increases with the extension of working hours, and the resulting job pressure will affect their health, and even their safety. We verified that both salary and working hours have a moderating effect on labour intensity, and the effect of working hours is greater, so Hypothesis 2 is tested.

## Discussion

**Figure 1:**
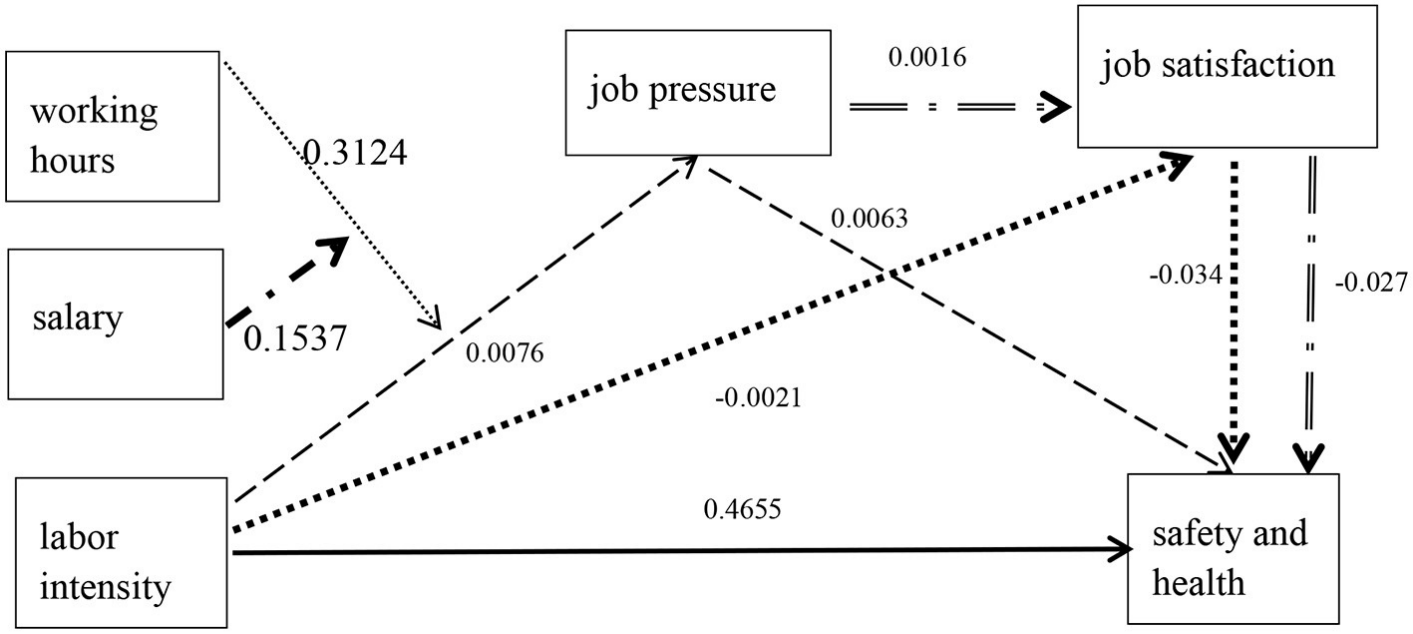
Impact path of the labour intensity on safety and health.

We can see the health mediating effect of IDS riders in Figure 1. Overall, in the mediating effect, job pressure has not only a strong impact on the front-end effect but also a strong mediation path in the back-end effect. Job satisfaction is a weak mediation path in both the front-end effect and the back-end effect. The mediating effect of job pressure is 0.1562 (β_1_-β_3_ = 0.4655–0.3093). The change in salary causes the change in working hours, which leads to the change in labour intensity and job pressure. The moderating effect of salary is 0.1537 and the moderating effect of working hours is 0.3124. Working hours increase labour intensity and then affect safety and health through job pressure. Labour intensity has a positive impact on safety and health. The total effect of labour intensity on safety and health is 0.4655.

The index of job pressure mainly includes five dimensions: “complete rest days per week, signing of the labour contract, social security participation, perception of competitive pressure, and perception of dealing with bad comments.” Take the mean value as the working pressure variable and evaluate it with 4 grades of “very small, small, general, large, or very large.”

The index of job satisfaction is formed by a six-factor analysis of “organisation satisfaction, management satisfaction, job reward satisfaction, working atmosphere satisfaction, the job itself satisfaction, and human resource management satisfaction.” Five grades are formed by Likert five subscale methods: “very dissatisfied, relatively dissatisfied, average, relatively satisfied, and very satisfied.”

The regression coefficient of labour intensity is 0.0076, which can cause work pressure. Labour intensity can directly lead to the decline of physical health and make IDS riders enter a state of overwork. For other job characteristics, it appears that workers in the app-enabled gigs are ordinarily doing standardised tasks repeatedly over a period of time, the structured algorithmic management techniques offer workers a high level of autonomy (50). Unstable employment tends to negatively affect health status. As it causes psychological and physical health risks, such as low mental health, dissatisfaction with physical health, anxiety, or high blood pressure. Platforms have added “digital reputation mechanisms” or “evaluating and rewarding mechanisms.” Their real motivation is to let IDS riders grab orders and compete to increase labour intensity.

The regression coefficient of working hours is 0.3124, which has the greatest impact on labour intensity. The survey found that 14.2% of IDS riders worked more than 12 h. In European countries, around 80% of platform workers declare platform work to be a secondary or tertiary source of income (51, 52). Even when platform work is the main source of income, the number of working hours is often low. Platform workers typically work low numbers of hours per week in European countries. A positive association with the number of hours worked per week was found for traffic penalties (or fines) (53). Working for a long time not only affects the health of IDS riders but also endangers their life safety.

The influence of salary is two-way, with both income effect and substitution effect. This study found that the income effect of IDS riders is still dominant in China. Although some studies have used evidence to show that piecework wage in a casual economy increases health (54). But at the same time, some studies have shown that performance-based pay is harmful to health. Vietnamese garment factories and American shoe factories that implement performance-based pay have had poor physical conditions and emotional health (55). The present study corroborates the UK findings for US workers, with poorer health outcomes reported for piece-rate workers than for salaried workers (56). Giggers often earn below minimum wage without any entitlements to social benefits (57–62), and prices are being raised or decreased by an algorithm depending on demand (63). Poor health outcomes linked to performance and piece-rate might further erode a company's bottom line.

Under current law, it is impossible for all the IDS riders to pay workers' compensation insurance for platform workers. Therefore, it is difficult for platform workers to manage chronic diseases, work-related diseases, and occupational diseases due to difficulties in health examination and health care.

## Limitations and Future Research Directions

First, our study results are limited to a sample of IDS employees currently working in two big cities in China, which has a more formal employment setting as reflected by the large proportion of IDS workers in the country. China has a vast territory and IDS business is generally developing, IDS in smaller and medium-sized cities should have more characteristic problems to be resolved. As such, our results should be generalised with caution to broader contexts.

Second, there is no heterogeneity analysis for IDS riders in this paper. After all, few IDS riders are female. Only 2.5% of the female respondents in our questionnaire, the analysis of the difference between male and female groups can not reflect the truth. Another reason is that women may be slightly poorer than men in security and cycling technology. They are not as fast as men in grabbing orders, and their orders are less than men. So, their labour intensity may be weaker than that of men, and the security of female IDS riders is relatively higher.

Third, the research deficiency is that the variation is not examined when an order was completed in unit time. The algorithm of time compression has become a trick to improve labour intensity in the AI system. After comparing the labour intensity between a normal speed and a limited speed, we may portray the mechanism of labour intensity to improve. Future research will collect the average speed in an order to make up for this deficiency.

## Theoretical Contributions

This study makes two key contributions to the knowledge base around the IDS riders. First, based on the life cycle theory of labour supply decision-making, a two-stage decision-making analysis is carried out on the consciousness of labour intensity. Work and safety and health are in two periods for decision-making, so this decision-making view should contribute to the progress of the traditional employment theory which is the basis of stable employment and high-quality employment.

Second, this study represents a pioneering attempt to analyse the theoretical explanation of labour control. The work of IDS is seemingly free, while it is actually a non-free labour process controlled by the platform. This study explains the deep reason why IDS appears seemingly free, but actually has no freedom from the comparison of substitution effect and income effect. The work caused by the substitution effect will make IDS riders lose their freedom as long as they start to work. IDS riders only have the freedom to choose to work or not, and never to work.

## Practical Implications

This study found that the job pressure comes from the labour intensity. Different from the traditional method, labour intensity comes from the work overweight by the management in traditional employment. While IDS riders increase their labour intensity without much prompting. They extend their working hours just for the temptation of salary, resulting in overwork. They lose their freedom to work in the game in order-grabbing which is seemingly free. Under the pressure of assessment, IDS riders deliver an order in a shorter and shorter time. Though IDS riders want to earn quick money under the order reward mechanism, the company should rely on the obligation specification following the labour law to cultivate employees' professional commitment, rather than only on the monetary benefits.

More and more orders to deliver, less and fewer rights to choose. The delivery sequence and riding route are locked and monitored by the platform. They even have no autonomy to deal with physical discomfort, accidents, rainstorms, and other special work environments. The labour intensity of IDS riders is increasing with their overwork, and so does the job pressure which is caused by the assessment. Both of them are not beneficial to IDS riders. To reduce the negative economic losses caused by IDS riders' safety and health issues, the company should formulate a working algorithm within a reasonable labour intensity.

In terms of their behaviour, it is very dangerous to watch their mobile phones while their riding. In particular, keep safety riding in an extreme work environment. In addition to providing a “health bag” and other necessary equipment, we should pay more attention to the IDS riders' rights. They should also be given the right to keep safe and healthy.

## Conclusion

In this paper, an intertemporal utility model is established to measure the labour intensity of IDS riders. An IV-Logit Model is used to investigate the impact of the change in labour intensity on IDS riders' safety and health combined with the survey data. Then the mediation path of job pressure is analysed and explored. The following conclusions and understandings are obtained:

(1) Hypothesis 1 is supported clearly by regression. The labour intensity has a positive impact on safety and health. The labour intensity increases, and the risk index of IDS riders' safety and health increases.

(2) Salary level and working hours have a moderating effect on labour intensity, so Hypothesis 2 is tested to be true. To the two moderating variables, the moderating effect of working hours is greater, and the moderating effect of salary is mainly determined by the total effect between the income effect and the substitution effect. As for IDS riders, their substitution effect is greater than their income effect.

(3) Predicted paths are not completely supported in Hypothesis 3. Job satisfaction is not effectively supported, while job pressure is the dominant mediation in the mediation paths. The increase in job pressure has a significant positive impact on the level of overwork in safety and health.

Piece rate pay designed to promote efficiency may have important negative implications for worker health, especially for the most vulnerable members of the US workforce (64), such as women, minorities, and low-income workers. Given the growing popularity of performance-based pay in the gig economy, more research is needed to determine if the practise is justified from a public health perspective (65). McDowell et al. suggested that vulnerable jobs include job-related insecurity, lack of legal rights and labour rights, lower salary levels, and higher occupational health risks (66). The work design in the IDS platform company is from the algorithms (67). Undeniably, the algorithms could not provide high-quality employment for IDS riders. IDS riders are more vulnerable.

The IDS riders should be put in a reasonable salary and working hours system to keep healthy. As no basic salary guarantee and appropriate working hours were designed in the IDS platform economy, it would be unrealistic that IDS riders reduced labour intensity.

It is never the normal way to improve the salary depends on grabbing more, riding faster. Undoubtedly, active overwork under the high pressure of work and life should damage their health. In particular, it is very dangerous for IDS riders being busy watching or grabbing orders while they are riding, especially in the mood of fatigue. The platform economy is recommodifying labour. We need to democratise it (68).

The findings above have obvious reference value for promoting the development of high-quality employment in China. Firstly, nearly 3 million workers are employed, and the employment group of IDS riders is expanding. Only IDS riders are safe and healthy, and the structure of whole social employment will be healthy. Secondly, IDS riders are controlled by the impersonal platform algorithm systems. In the current new economic policies, we should be more vigilant about labour intensity to be improved by the economic effects of algorithms and artificial intelligence technology. Finally, to study the safety and health of IDS riders in economic theory, an Intertemporal Utility Model was constructed using C-D Function. With the introduction of the intertemporal concept, we overcome the shortcomings of previous research on IDS riders in economic theory. The intertemporal concept can prevent us from studying the shortsighted behaviour of IDS riders.

Key evidence has surfaced since Adam Smith's early conjecture to support his theory of the negative health impacts. Taylorism has been largely criticised for turning workers into an automaton or machines who fail to find meaning in their work.

To cope with the new occupational health and safety issues, we need to establish new concepts of “decent work” and standardise regulations, which are responsible for health and safety (69). With the vigorous development of the platform economy, the country should pay more attention to vulnerable employees, as well as high-quality employment.

## Data Availability Statement

The raw data supporting the conclusions of this article will be made available by the authors, without undue reservation.

## Ethics Statement

Ethical review and approval was not required for the animal study because IDS riders' employment qualification meets the constraints of labor law. In line with the legal age of 18, there are no inhumane moral problems such as employing child labor.

## Author Contributions

TC is responsible for research ideas and overall research work. DT conducts the investigation for data. PD corrects the grammatical errors in the article. EZ and JH sort out the data and materials. All authors contributed to the article and approved the submitted version.

## Funding

We thank the Graduate Teaching Reform Project “Prevention and Handling of Labor Disputes in China's First Labor Court” and the program “Early Warning of Labor Relations in the New Stage: prevention based on sudden public crisis” (31512211014) for giving financial support.

## Conflict of Interest

The authors declare that the research was conducted in the absence of any commercial or financial relationships that could be construed as a potential conflict of interest.

## Publisher's Note

All claims expressed in this article are solely those of the authors and do not necessarily represent those of their affiliated organizations, or those of the publisher, the editors and the reviewers. Any product that may be evaluated in this article, or claim that may be made by its manufacturer, is not guaranteed or endorsed by the publisher.

## Footnotes

^1^In 2014, the State Council issued the criteria for the division of city size, which divides cities into categories based on the permanent resident population in urban areas. Cities with a permanent resident population of more than 5 million and less than 10 million are megacities, and cities with a permanent resident population of more than 10 million are megacities. According to the data of the seventh national census, the total population of Beijing in 2021 is 21893095. By the end of 2021, Wuhan had a permanent resident population of 13.6489 million, which is less than the threshold of 50000 super cities.

^2^Two factor analyses were conducted, and six characteristic factors were extracted for the first time in 1970. The second analysis was attempted in 1985. The second analysis was a factor analysis of 81 items. The data used in factor analysis are the data of 11897 employees in the motor equipment manufacturing industry, including 8888 men in 91 workplaces and 3009 women in 32 workplaces.

^3^The standard is generally accepted by Chinese scholars. Such as Huang et al. (46–48).

^4^8 departments: Ministry of Human Resources and Social Security, National Development and Reform Commission, Ministry of Transport, Emergency Management Department, General Administration of Market Supervision, State Medical Insurance Bureau, Supreme People's Court and All China Federation of Trade Unions.

^5^The calculation formula of labor intensity index is as follows: I = 3T + 7M. Where: I - labor intensity index; T - labor time rate = net labor time in working days (minutes) / total working hours in working days (minutes) (%); M-8 hour working daily energy metabolism rate (kJ / min · m2); 3 - calculation coefficient of labor time rate; 7 - calculation coefficient of energy metabolic rate.

^6^0-6 represents the labor intensity of different “Daily Distance ”(6 levels is divided in this paper).

^7^It is a constant greater than 0 because IDS riders are homogeneous, and the parameters have no difference in different IDS riders.

^8^As the moderating variable of salary and working hours, *G*_*ij*_will be carried out hierarchically during regression.
